# Retrospective Multicenter Real-Life Study on the First-Line Treatment of Classical Hodgkin Lymphoma in Argentina

**DOI:** 10.1007/s44228-022-00008-4

**Published:** 2022-06-22

**Authors:** Carolina Mahuad, Otero Victoria, Korin Laura, Martinez Enriqueta, Warley Fernando, García Rivello Hernán, Cristaldo Nancy, Kohan Dana, Zerga Marta, Garate Gonzalo, Vicente Repáraz María de los Ángeles, Aizpurua Florencia, Rojas Bilbao Erica, Cerana Susana, Funes Maria Eugenia, Plaza Iliana, Foncuberta Cecilia, Vijnovich Baron Anahí, Cranco Santiago, Vitriu Adriana, Gomez Mariela, Lavalle Justina, Casali Claudia, Clavijo Manuela, Melillo Luciana, Cabral Lorenzo Maria Cecilia, Miroli Augusto, Fischman Laura, Pavlove Maximiliano, Miodosky Marcela, Cugliari Silvana

**Affiliations:** 1 Servicio de Hematología, Hospital Alemán, CABA, Pueyrredón 1640, CP 1118 Buenos Aires, Argentina; 2 Servicio de Hematología, Hospital Italiano, Buenos Aires, Argentina; 3 Servicio de Hematología, Instituto Alexander Fleming, Buenos Aires, Argentina; 4 Servicio de Hematología, Hospital de Clínicas “José de San Martín”, Buenos Aires, Argentina; 5 Hospital Británico, Rosario, Argentina; 6 Servicio de Hematología, Instituto de Oncología “Ángel Roffo”, Buenos Aires, Argentina; 7 Servicio de Hematología, Hospital Churruca, Buenos Aires, Argentina; 8 Servicio de Hematología, Hospital Durand, Buenos Aires, Argentina

**Keywords:** Hodgkin disease, Survival, Real world evidence, Toxicity, Pet-adapted

## Abstract

There are no data in Argentina on the response rates to first-line treatment of classical Hodgkin Lymphoma (cHL) outside clinical trials. A total of 498 patients from 7 public and private hospitals in Argentina were retrospectively examined. The median follow-up was 37.4 months (CI 95% 17.7–63.5). The median time from diagnosis to treatment was 22 days (IQR 14–42), which was significantly longer in public hospitals (49.3 (IC 95% 38.5–60.2) versus 32.5 (IC 95% 27–38); *p* = 0.0027). A total of 96.8% of patients were treated with ABVD.:84.3% achieved complete remission (CR) and 6.02% partial remission (PR), being the CR rate higher in private hospitals. End-of-treatment metabolic CR was achieved in 85.4% (*n* = 373). The interim PET scan was widely used in our cohort (70.5%; *n* = 351), but in only 23.3% (*n* = 116) was the treatment strategy response-adapted. The 5-year progression-free survival (PFS) was 76% (CI 95% 70–81). The 2 and 5-years-OS rates were 91% (CI 95% 88–94%) and 85% (CI 95% 80–89%), respectively. No differences in OS were found between public and private institutions (*p* = 0.27). This is one of the largest retrospective cHL cohorts reported. In Argentina ABVD is the chemotherapy regimen of choice and, although it is well tolerated, it is not exempt from toxicity. We showed that early initiation of treatment impacts the induction results. Although the use of PET scan is widespread, only a minority of patients was treated with respons- adapted strategies. The use of PET-guided treatment is strongly encouraged.

## Introduction

Hodgkin lymphoma is an aggressive hematological malignancy with high cure rates [1]. It is most commonly diagnosed in young adults, but has a bimodal age distribution curve, with up to a quarter of cases presenting in patients aged 60 and above [2]. In the past few decades, the survival of patients treated for classical Hodgkin Lymphoma (cHL) has improved dramatically, as a result of the development of multiagent chemotherapy, more accurate radiotherapy, and enhanced possibilities to treat complications during and after treatment [3]. The latest estimated incidence of cHL in Argentina is 842 cases/year [4]. However, there are no local data on response rates (RR) to first-line treatment. The, Grupo Argentino de Tratamiento de Leucemia Aguda (GATLA) cooperative group reported 3-year progression free survival (PFS) and overall survival (OS) rates, regardless of stage, of 90% and 98%, respectively, following a PET-driven therapeutic approach [5].

Furthermore, given that cHL patients have a long-life expectancy following first-line treatment, the risk of acute and, specially, long-term treatment-related toxicity is an aspect of particular concern [3] that has not been addressed in Argentina.

Although cHL has a high cure rate, 10% of the patients are primary refractory. Around 30% of them relapse after achieving complete remission (CR), resulting in an estimated 5-year OS around 90% in stage I–IIa and 60% in stage IV [2].

The primary objectives of this study were to assess the real-world RR, PFS and OS, after first-line treatment of cHL in public and private hospitals in Argentina. Secondly, we sought to identify epidemiological characteristics of the patients of the participating hospitals that could determine discrepancies in the response to first-line treatment.

## Materials and Methods

Data from all consecutive newly diagnosed cHL patients aged 18 and above who were diagnosed and treated in eight public and private participating medical centers of Argentina (Hospital Alemán, Hospital Italiano, Hospital de Clínicas José de San Martín, Instituto de Oncología Ángel Roffo, Instituto Alexander Fleming, Hospital Durand, Hospital Churruca- Buenos Aires and Hospital Británico- Rosario) between 1/1/2008 and 2/1/2019 were retrospectively reviewed. The study was approved by the local Institutional Committees. The inclusion criteria for this study were: age 18 or above at diagnosis, histologically confirmed cHL, treatment with chemotherapy and available follow up data. Baseline characteristics, treatment protocol, adverse events during and after treatment and outcome measures were recorded. PET-CT results at diagnosis, interim analysis (iPET-CT), and end-of-treatment (EOT-PET-CT) were recorded and analyzed. Following local treatment protocols in the participating sites, iPET-CT was scheduled for after 2 cycles of chemotherapy, 12–14 days after the last chemotherapy administration. The 5-point scale Deauville score (DS) of the level of residual FDG uptake at compromised sites was used to evaluate response to treatment on iPET and EOT-PET-CT scans [6, 7]. DS of 1–3 on iPET-CT was considered negative. For all cases which were treated before the incorporation of the DS, the imaging was reviewed at the institution, and the DS was applied to evaluate response. iPET-CT was not available in all institutions. In the latter cases, a CT was performed. The 2014 Lugano classification was used for staging and assessment of response to treatment [7], and the prognostic groups were defined according to the German Hodgkin Study Group (GHSG) [8, 9].

Statistical analysis was carried out with Stata 16.1 (StataCorp LLC). Descriptive statistics was used to analyze histopathological findings and clinical variables. *t* tests were used to compare means of normally distributed variables between two groups. Proportions across categories were compared using chi-squared tests. PFS was defined as the time from diagnosis of cHL to death or disease relapse/progression, including less than complete remission (CR) at the end of the treatment protocol. OS was defined as the time from diagnosis of cHL to death or last follow-up visit. Qualitative variables were expressed as total number and percentage (%) and quantitative variables as median and interquartile range (IQR). Survival rates were estimated by the Kaplan–Meier method and compared by the log-rank test. Variables with *p* value < 0.05 in univariate analysis were entered into the multivariable Cox proportional hazards model in a stepwise fashion. A *p* < 0.05 was established as limit of significance for all analyses.

## Results

Five hundred and twenty patients from seven public and private hospitals in Buenos Aires and Rosario were examined. Twenty-two patients (22/520) had nodular lymphocyte predominant Hodgkin Lymphoma and were excluded form this analysis; only data on the 498 patients with cHL are included in this study.

The baseline characteristics of patients are presented in Table 1. Overall, the median age at diagnosis was 34.5 years (interquartile range (IQR), 25–54). A male predominance was observed (*n* = 281, 54%). Most patients had nodular sclerosing subtype (*n* = 364, 73%) and B symptoms (*n* = 294, 56.65%).

**Table 1:** Characteristics of patients diagnosed with Classical Hodgkin lymphoma (cHL) treated in first line

Number of patients	498
Age, Median (IQR)	34.5 (25–54)
Male/Female (%)	281 (54)/239 (46)
Stage (%)	
1	4.81
2	47.31
3	19.23
4	28.65
Bulky disease yes/no (%)	164/356 (31.5/68.5)
Extranodal involvement yes/no (%)	173/347 (33.3/66.7)
B Symptoms yes/no (%)	294/225 (56.65/43.35)
Risk group (%)	
Early favorable	15.77
Early unfavorable	36.35
Advanced favorable	15.57
Advanced unfavorable	32.31
Charlson score, Median (IQR)	2 (0–2)

Only a minority had bulky disease (*n* = 164, 31.5%), defined as a tumor mass of > 7 cm and, extranodal involvement (*n* = 173, 33.3%). A low proportion of patients were stage I at diagnosis (4.8%), while almost half of the cohort were at stage II (47.3%). Forty eight percent of our patients presented with advanced disease (stage III and IV, 19.23% and 28.65%, respectively). Regarding risk stratification, early and advanced favorable groups represented around 15% of the patients each, while around 30% were classified as early unfavorable or advanced unfavorable (Table 1).

**Fig. 1:**
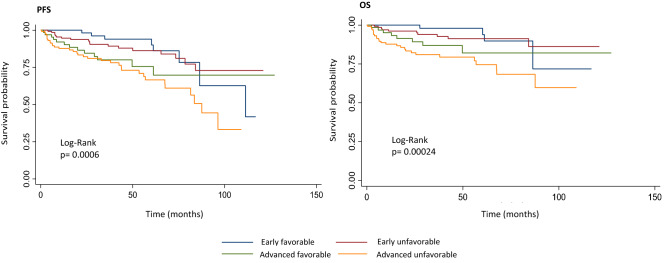
Progression Free- and Overall Survival by risk group

**Fig. 2:**
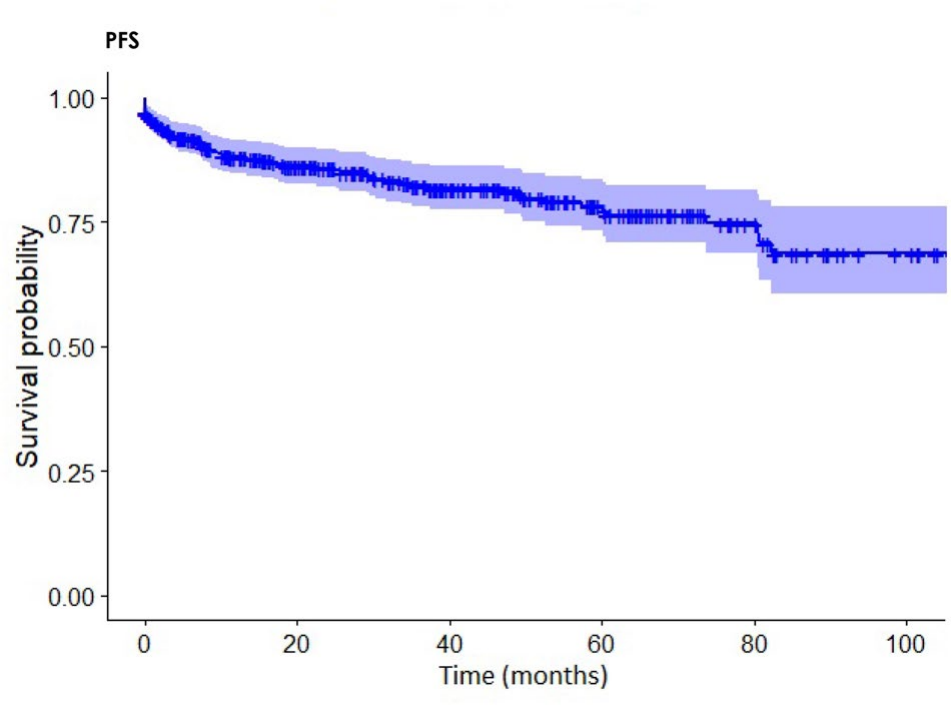
Progression Free Survival in the complete cohort of patients

The median follow-up time was 37.4 months (CI 95% 17.7–63.5). The median time from diagnosis to treatment was 22 days (IQR 14–42), and was significantly longer in public hospitals (49.3 (IC 95% 38.5–60.2) versus 32.5 (IC 95% 27–38); *p* = 0.0027). The therapeutic strategies and outcome in the full cohort and according to risk group is shown in Table 2. The majority of patients received ABVD (adriamycin-bleomycine-vinblastine and dacarbazine) (96.8%) as first-line treatment. In 17.1% of patients, a dose modification or temporary drug suspension was required due to hematological toxicity and neutropenic fever in most of the cases; the majority (83%) received all cycles as planned (Tables 2 and 3). A total of 84.3% of patients achieved CR and 6.02% PR, being the CR rate higher in private hospitals. Of 498 patients, 306 (61.5%) received treatment in private institutions. Among these, 265 (86.6%) achieved CR; 8 (2.6%) PR and 33 (10.8%) were primary refractory. One hundred ninety-two (38.5%) patients received treatment in public hospitals. Of these, 192 (78.6%) achieved CR after first-line of treatment; 23 (12%) PR, and 18 (9.4%) were primary refractory. The CR rate was higher in private hospitals (86.6% versus 78.6% *p* = 0.0001). Additionally, an estimated 9.64% of the full cohort of patients had progressive disease (PD) at the end-of first-line treatment (Table 2). With respect to PET responses, 85.3% (*n* = 373) had negative (DS1-3) EOT-PET-CT results. IPET-CT was performed in 70.5% of our cohort (*n* = 351), with 83.8% achieving metabolic CR. However, only 23.3% (*n* = 116) were treated with response-adapted strategies (6.5% deescalated to AVD). Regarding hematologic toxicity, anemia was present in 28.2%, neutropenia in 56.4% and thrombocytopenia in 7% of patients (Table 3). Twelve patients presented febrile neutropenia during treatment. With respect to non-hematological toxicities, 28.9% of patients experienced adverse events (56/144 were recorded as pulmonary toxicity) (Table 3). No cases of death due to acute treatment-related toxicity were registered. Primary refractory disease was reported in 48 patients (9.64%), and 69 (14%) relapsed during follow-up, being the median time to relapse 4.4 months (CI 95% 0–13). During the follow-up period, 60 patients died (12.04%). Among these, death was due to lymphoma progression in 32 (53.33%), and to therapy-induced toxicity in 26 (44%) (255 infection, 12% pulmonary toxicity and 7% other) (Table 3). Regarding the age of deceased patients, it was significantly higher (Mean age 53 years-old [CI 95% 48–58.5] vs 38 [CI 95% 37–40]; *p* < 0.0001). The mean Charlson’s comorbidity index was 3.9 (IC 95% 3.1–4.8) for deceased patients versus 1.8 (IC 95% 1.6–2.02); *p* < 0.0001 for the alive patients. The 5-year PFS was 76% (CI 95% 70–81). The 2 and 5-year OS rates were 91% (CI 95% 88–94%) and 85% (CI 95% 80–89%), respectively (Figs. 1 and 2: PFS and OS according to risk group and PFS for the full cohort). No differences in OS were found between public and private institutions (*p* = 0.27).

**Table 2:** Therapeutic strategy and outcome according to risk group and, in the full cohort

	Chemotherapy protocol *n*/total (%)		I-PET CT Deauville Score (1–5) *n*/total (%)	EOT-PET CT Deauville Score (1–5) *n*/total (%)	Pet-adapted treatment *n*/total (%)	Adequate cycling *n*/total (%)	Response to treatment *n*/total (%)
Early favorable *n* = 78	ABVD	76/78(97.4)	1 = 26/52 (50)	1 = 48/77 (62.3)	Y: 14/78 (18)	Y: 70/78 (90)	CR: 74/78 (95)
	AVD	2/78 (2.6)	2 = 18/52 (34)	2 = 21/77 (27.3)	N: 64/78 (82)	N: 8/78 (10)	PR: 3/78 (3.7)
	E-BEACOPP		3 = 4/52 (8)	3 = 3/77 (3.9)			SD: 0/78
	Other		4 = 4/52 (8)	4 = 4/77 (5.2)			PD: 1/ 78 (1.3)
			5 = 0	5 = 1/77 (1.3)			
Early unfavorable *n* = 181	ABVD	181/181(100)	1 = 47/140 (33.6)	1 = 88/167 (52.7)	Y: 47/181 (26)	Y: 157/181 (87)	CR: 159/181 (87.86)
	AVD		2 = 52/140 (37.2)	2 = 48/167 (28.7)	N: 134/181 (74)	N: 24/181 (13)	PR: 10/181 (5.52)
	E-BEACOPP		3 = 23/140 (16.4)	3 = 9/167 (5.4)			SD: 0/181
	Other		4 = 17/140 (12.1)	4 = 14/167 (8.4)			PD: 12/181 (6.62)
			5 = 1/140 (0.71)	5 = 8/167 (4.8)			
Advanced favorable *n* = 78	ABVD	77/78 (98.7)	1 = 14/52 (27)	1 = 24/63 (38.1)	Y: 19/78 (24)	Y: 65/78 (83)	CR: 67/78 (85.91)
	AVD		2 = 17/52 (32.7)	2 = 23/63 (36.5)	N: 59/78 (76)	N:13/78 (17)	PR: 2/78 (2.56)
	E-BEACOPP		3 = 8/52 (15.4)	3 = 5/63 (8)			SD: 0/78
	Other	1/78 (1.3)	4 = 12/52 (23)	4 = 6/63 (9.5)			PD: 9/78 (11.53)
			5 = 1/52 (1.9)	5 = 5/63 (7.9)			
Advanced unfavorable *n* = 161	ABVD	148/161(91.92)	1 = 31/107 (29)	1 = 64/130 (49.2)	Y: 36/161 (22)	Y: 121/161 (75)	CR: 120/161 (74.53)
	AVD	2/161(1.24)	2 = 35/107 (32.8)	2 = 32/130 (24.6)	N: 125/161 (78)	N: 40/161 (25)	PR: 15/161 (9.32)
	E-BEACOPP	5/161(3.12)	3 = 19/107 (17.7)	3 = 8/130 (6.1)			SD: 0/161
	Other	6/161(3.72)	4 = 20/107 (18.7)	4 = 9/130 (7)			PD: 26/161 (16.15)
			5 = 2/107 (1.8)	5 = 17/130 (13.1)			
Full cohort *n* = 498	ABVD	482/498 (96.8)	1 = 118/351 (33.6)	1 = 224/437 (51.2)	Y: 116/498 (23.3)	Y: 413/498 (83)	CR: 420/498 (84.34)
	AVD	4/498 (0.80)	2 = 122/351 (34.75)	2 = 124/437 (28.4)	N: 382/498 (76.7)	N: 85/498 (17)	PR: 30/498 (6.02)
	E-BEACOPP	5/498 (1.00)	3 = 54/351 (15.4)	3 = 25/437 (5.7)			SD: 0/498
	Other	7/498 (1.40)	4 = 53/351 (15.11)	4 = 33/437 (7.6)			PD: 48/498 (9.64)
			5 = 4/351 (1.14)	5 = 31/437 (7.1)			

*iPET CT* interim PET CT, *EOT-PET CT* end-of-treatment PET CT, *Y* yes, N = no, *CR* complete response, *PR* partial response, *SD* stable disease, *PD *progressive disease

**Table 3:** Treatment related toxicity and causes of death

Hematological toxicity *n*/total (%)		Non-hematological toxicity *n* (%)	Causes of death *n* (%)
Anemia	138/498 (28.2)	Allegy, any 9 (1.8) Phlebitis 22 (4.42) DVT 10 (2) Vasculitis 2 (0.4)	Total of deaths 60/498 (12.04) Cardiovascular 1/60 (1.67) Disease progression 32/60 (53.33) Other cancers 1/60 (1.67) Infection 15/60 (25) Death-inducing pulmonary toxicity 7/60 (11.67) Other 4/60 (6.66)
Neutropenia	281/498 (56.4)		
Thrombopenia	35/498 (7.0)	Gastrointestinal, all 40 (8) Mucositis 3 (0.6) Nausea/vomiting 27 (5.4) Diarrhea 3 (0.6) Hepatitis 4 (0.8) Obstipation 3 (0.6) Acute renal failure 2 (0.4) Neuropathy 7 (1.4)	
		Infections, all 26 (5.22) Pneumonia 6 (1.2) Invasive fungal infections 1 (0.2) Catheter infection 3 (0.6) TBC reactivation 1 (0.2) Disseminated HZV infection 1 (0.2) Cellulitis 1 (0.2) Cholecystitis 1 (0.2) Febrile neutropenia 12 (2.4)	
		Cardiovascular, all 4 (0.8) ACS 4 (0.8)	
		Pulmonary, all 56 (11.24) Pneumonitis 2 (0,4) BOOP 1 (0.2) Fibrosis 2 (0.4) Other, not specified 51 (10.24)	

*DVT* deep vein thrombosis, *BOOP* bronchiolitis obliterans organizing pneumonia, *TBC* tuberculosis, *HZV* herpes zoster virus, *ACS* acute coronary syndrome

Variables associated with significantly better outcomes were (Table 4): age younger than 60, the absence of extranodal disease or risk factors such as leukocytosis, lymphopenia and hypoalbuminemia. A survival advantage was also found in patients with normal ESR, stage I-III, early favorable and advanced favorable stages, Charlson score < 3 (*p* < 0.0001) and for those who achieved a negative EOT-PET CT. Variables that remained independent predictors of OS on multivariate analysis were the Charlson score < 3 (HR of 1.4 (CI 95%1.1–1.7; *p* = 0.001) and the EOT-PET-CT (HR of 2.3 (CI 95% 1.7–3.2; *p* < 0.0001).

**Table 4:** Univariate and Multivariate analysis

	Univariate analysis		Multivariate analysis	
	HR^a^ (IC95%)	*p*	HR^a^ (IC95%)	*p*
Age	1.0 (1.03–1.06)	**0.0001**	1.0 (0.95–1.02)	0.36
Sex (Female)	1.5 (0.8–2.7)	0.151		
Extranodal disease	2.2 (1.2–3.8)	**0.0082**	2.5 (0.68–9.06)	0.16
Bulky disease	1.5 (0.9–2.8)	0.143		
Stage	1.7 (1.3–2.3)	**0.001**	0.8 (0.37–1.62)	0.50
Lymphopenia	3.1 (1.7–5.8)	**0.0001**	0.9 (0.2–3.59)	0.90
Leukocytosis	0.7 (0.3–1.7)	0.438		
Hypoalbuminemia	3.0 (1.5–6.1)	**0.002**	1.6 (0.55–4.49)	0.39
High ESR^b^	2.1 (1.1–3.9)	**0.025**	1.1 (0.35–3.58)	0.83
Unfavorable risk	3.4 (1.2–9.3)	**0.020**	1.2 (0.8–1.8)	0.44
Charlson score < 3	1.3 (1.2–1.4)	**0.0001**	1.4 (1.1–1.7)	**0.001**
EOT-PET CT^c^	2.1 (1.6 to − 2–7)	**0.0001**	2.3 (1.7–3.2)	**0.0001**

^a^Hazard Ratio

^b^Erytrocyte Sedimentation Rate

^c^End-of-treatment PET CT

## Discussion

Since a real-world analysis was lacking, we retrospectively assessed a large Argentinian cohort of cHL patients who underwent first-line treatment outside of clinical trials. Ours is one of the largest retrospective cohorts reported in cHL and, importantly, reflects a real-life experience outside a clinical trial setting. Previous studies have shown discrepancies in outcome of cHL patients treated in a clinical trial [10], or in a cancer center [11] as compared to those treated in local community hospitals. We found similar epidemiological characteristics, RR, PFS, and associated variables to those from other series [12–16]. Nevertheless, the 5-year OS in our cohort was higher than previously reported. Bouliotis et al.reported the survival time, excess mortality and cure fraction of patients from the Nottinghamshire Lymphoma Registry relative to the English and Welsh general population, and also compared disease trends during the 70 s, 80 s and 90 s. The relative survival probabilities at 10 years increased progressively in the different periods: 52.3% for the 1973–1982 cohort, 67.8% (1983–1992) and 75.7% (1993–2002) [17]. A national French study analyzed survival for various cancers using registry data from 16 regions [18]. Using the Pohar Perme model, they reported a 10 -year age-standardized net survival (deaths only from HD) for 2260 patients diagnosed between 1989 and 2004, as being about 80% for men (77–83%), 73% for women (70–77%) and 76% (73% to 78%) for both genders. In an analysis from the Swedish National Cancer registry of 6949 patients with HD (1973–2009) the 10-year relative survival of patients in the 36 to 50-year age group improved during the study period: 57% (1973–1979), 71% (1980–1986), 80% (1987–1993), 93% (1994- 2000) and 93% (2001–2009) [19]. A more recent study by Bröckelmann et al. evaluated 10 meta-analyses, including 89 randomized and controlled trials, and 81 prospective or retrospective trials published between 2012 and 2017, and showed that the 5 five-year survival of patients with Hodgkin lymphoma was 95% [20]. The latter study shows outcomes more similar to ours, and probably reflects the risk- and response-adapted strategies implemented in the last decade.

As to the differences we found between public and private hospitals, it must be highlighted that, in Argentina, access to diagnostic and therapeutic tools tends to be faster in private institutions. In our study, patients treated in the latter achieved CR in a higher proportion. It is noteworthy that achieving higher rates of CR, as it was found in private hospitals compared to public institutions, was not translated in an improvement of OS. This may be due to the low percentage of patients who progress, and also probably to the success of the available rescue strategies for patients who are in first relapse or who progress after first-line [20–26].

A finding in our cohort that has not been reported in other studies, which perhaps impacts the possibility of achieving CR, is the time between diagnosis and initiation of first-line treatment. This was significantly shorter in private institutions (22 versus 49 days; *p* 0.0027), where the percentage of patients achieving CR after first-line treatment was significantly higher (86.6% versus 78.6% *p* = 0.0001).

ABVD is the preferred chemotherapy regimen in Argentina and, as our study shows, it is well tolerated but not exempt from toxicity. The toxicity spectrum found in our study is similar to that previously reported in the literature [16].

Nowadays one of the challenges for patients with advance-stage Hodgkin´s lymphoma is to achieve high cure rates while lowering early and late treatment-related toxicity. The use of functional imaging with PET performed early in the course of therapy, offers a way to make treatment adjustments based on response to therapy. Recent studies showed excellent results by using PET2 to modulate therapy, with escalation for those with an unsatisfactory response and de-escalation for those with chemo-sensitive disease [27–30]. The RATHL (Response-Adapted Therapy in Advanced-Stage Hodgkin Lymphoma) trial showed that in PET2-negative patients, the bleomycin-deleted AVD was not inferior to ABVD. Omitting bleomycin reduced lung toxicity, with a 3-year PFS of 86%. In patients who were PET2-positive after two cycles of ABVD, changing to escalated BEACOPP improved outcomes, with a 3-year PFS rate of 71.1% and an OS rate of 82.8% [27].

PET scan was widely used to assess response in our cohort, but only a minority of 23.3% of the patients were treated with response adapted strategies. If we consider that in 44% of the patients, the main cause of death was therapy-induced (12% pulmonary toxicity), the use of PET-guided treatment should be strongly encouraged.

Within the main limitations of our study, we must mention its retrospective rather than prospective nature, and the lack of a central review of both the anatomopathological samples and PET images. Data collected through cancer registry analysis are dependent on accurate coding in the database. It is difficult to accurately track the number of chemotherapy cycles, PET scans, dosing, and duration of radiotherapy. The strengths of this analysis were the number of patients analyzed, the long follow-up and the uniform treatment in a multicenter setting, despite being outside a clinical trial.

It is possible that in the near future, the treatment of HL will change, with a new generation of drugs able to modify or even replace the current standards. This will doubtless represent a new challenge in the clinical management of this disease. Further research is needed to see if these novel drugs could improve the quality of life for both HL patients undergoing treatment and for the growing cohort of HL survivors.
